# The neurophysiological correlates of religious chanting

**DOI:** 10.1038/s41598-019-40200-w

**Published:** 2019-03-12

**Authors:** Junling Gao, Hang Kin Leung, Bonnie Wai Yan Wu, Stavros Skouras, Hin Hung Sik

**Affiliations:** 1 Buddhism and Science Research Lab, Centre of Buddhist Studies, The University of Hong Kong, Pokfulam, Hong Kong; 2 Department of Biological and Medical Psychology, Faculty of Psychology, University of Bergen, Bergen, Norway

## Abstract

Despite extensive research on various types of meditation, research on the neural correlates of religious chanting is in a nascent stage. Using multi-modal electrophysiological and neuroimaging methods, we illustrate that during religious chanting, the posterior cingulate cortex shows the largest decrease in eigenvector centrality, potentially due to regional endogenous generation of delta oscillations. Our data show that these functional effects are not due to peripheral cardiac or respiratory activity, nor due to implicit language processing. Finally, we suggest that the neurophysiological correlates of religious chanting are likely different from those of meditation and prayer, and would possibly induce distinctive psychotherapeutic effects.

## Introduction

Religious chanting is as common in Eastern cultures as praying is in Western. It is generally presumed that religious chanting can quieten fears and transcend the mind, thereby helping individuals to cope with hardship^1,2^. Nonetheless, the scientific studies on religious chanting are surprisingly scarce. The neurophysiological mechanisms underpinning the positive effect that religious chanting has been claimed to exert have not been illustrated by any decisive evidence. To fill this research gap, the present study combined advanced multi-modal electrophysiological and neuroimaging methods to investigate the neural correlates of chanting Amitābha Buddha^2^.

Chanting in faith of the Buddha Amitābha is the most widespread form of religious chanting and one of the oldest documented religious practices that are actively preserved to date^3,4^. It can be performed in a number of languages, including the ancient Indic Prakrit and Sanskrit, as well as Chinese and Japanese^5^. Based on the Mahayana Buddhist belief of the Western Paradise, monks and laity from different Mahayana schools traditionally chant the name of Amitābha Buddha as a daily ritual in order to be reborn in the Western Paradise, also referred to as the Pure Land. The chanting consists of repetitively reciting the few syllables comprising the name of Buddha Amitābha, either silently or aloud, as often as possible (typically at least for a few minutes per day) for arbitrary durations^6^. Performing the ritual in a quiet or isolated space is not mandatory and believers may even chant internally in public or while in transit, as long as the chanting is performed with sincere faith and focus. In essence, the philosophy of the Buddha Amitābha is easy and welcoming with a promise that all those who sincerely call upon this name will be reborn in his Pure Land, a philosophy that is reflected in the simplicity and accessibility of its main practice^7^. In Buddhist scripture, Buddha Amitābha is a fantastic figure associated with infinite light and infinite life^5^, as well as the creator of a world of equality and compassion, the Pure land, where all his believers and sentient beings can be reborn and saved in fair conditions that favor attaining enlightenment^6,8^. Although chanting regularly suffices for being considered a religious believer, according to prominent Buddhist thinkers, the longer and more intensely a believer engages in chanting Amitābha Buddha, the more probable it becomes to remain in sympathetic resonance with the Buddha’s pure mind and to be reborn in the Pure Land^8^. Chanting Amitābha Buddha faithfully on a long-term basis has been reported to be an effective meditational technique that can elicit blissful sensations and transcendental experiences^9^.

Being a religious meditative practice, religious chanting can be regarded as both meditation and prayer. The majority of relevant neurophysiological studies have focused on mindfulness meditation and have mainly demonstrated increased alpha and theta waves^1^. Very slow delta waves have been observed in a few EEG studies on mindfulness meditation without consistent findings^10^. A previous study on mindfulness meditation has shown that mindful breathing can induce coordination between the brain and heart activity^11^. The cardiac rhythm can be modulated by several factors including the sinus node activity, respiratory rate, and more importantly, the autonomic nervous system. Mindfulness training has been shown to improve cardiac sympatho-vagal balance^12^.

Up to date, very few EEG studies have been performed on the equally relevant practice of religious praying. For example, Doufesh and colleagues conducted a series of EEG studies on Muslim prayer, revealing increased alpha waves in occipital and posterior brain regions, as well as increases in high frequency (HF) of heart rate variability (HRV)^13^. However, the experimental paradigm employed featured resting state instead of a sham-prayer control condition, making it difficult to rule out potential confounding effects due to implicit language processing during prayer. More importantly, the EEG analyses employed did not feature source localization and thereby did not shed any light on specific brain regions involved in the neural processes under investigation.

With relatively recent advances in source localization, high-density EEG combines high temporal resolution with improved spatial resolution^14^. The traditional approach of comparing EEG results averaged across participants for each electrode channel poses potential problems in measuring signals from mixed sources or even different sources, across participants, due to variability in brain anatomy and electrode positioning. The alternative method of computing group statistics based on clusters of independent components of brain activity overcomes such limitations^15,16^. Through independent component analysis (ICA) the independent components (ICs; sometimes referred to as factors, latent variables or sources) are identified by minimising the mutual information of signal sources^15,16^. These components can then be grouped across participants via independent component clustering and statistical tests can compare the power of different frequency bands across conditions. Frequency bands in signal sources reflect rhythmic brain activity and can be informative of different states of neuronal activity or denote transitions between different mental states^17^. The present study aimed to adopt this methodology to illustrate the neural correlates of religious chanting.

We hypothesized that, similarly to meditation, repetitive religious chanting would lead to significant changes in brain activity and that such changes could be detected in sources of interest using high-density EEG and spectral analysis. Moreover, due to the positive emotions ascribed to Amitābha Buddha we expected affective changes to occur during religious chanting, accompanied by changes in peripheral physiological measurements, including the multi-band HRV indices and respiration rate.

## Results

**Figure 1:**
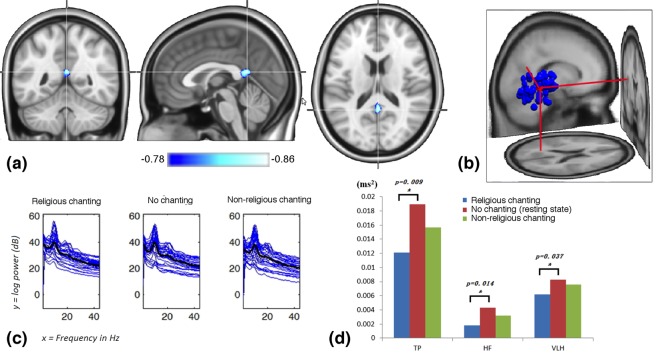
Multi-modal neuroimaging and electrophysiological results. Eigenvector centrality mapping applied on fMRI data, revealed that the posterior cingulate cortex is the area of the brain that decreased most in centrality, in a highly trained Buddhist meditator, during religious chanting compared to non-religious chanting. (**a**) EEG independent component clustering also revealed a cluster in the posterior cingulate cortex in an independent sample of 21 intermediate Buddhist meditators, during religious chanting. This cluster was selected for further analysis. (**b**) Spectrum analysis in the cluster of interest, followed by a one-way ANOVA and post hoc testing, revealed a significant increase of delta-band power during religious chanting, compared to non-religious chanting. (**c**) Compared to the no chanting resting state, religious chanting induced lower HRV total power (TP), as well as lower power in the high frequency (HF) and very-low-frequency (VLF) components of HRV (**d**).

The fMRI analysis suggested that the maximal difference in eigenvector centrality, during religious chanting compared to non-religious chanting, was in an approximately spherical cluster located in the posterior cingulate cortex (PCC) [volume = 81 mm^3^; radius ~3 mm; mean = −0.86; SD = 0.01; max = −0.87; peak MNI coordinates = “0 –51 19”; Fig. 1a]. This result was used to assess and confirm the soundness of the EEG independent component clustering, the results of which also featured a cluster in the PCC. Moreover, because the fMRI finding suggested that the PCC is the area decreasing most in centrality during religious chanting (in a highly trained meditator), the EEG IC cluster in the PCC was the cluster selected for more in-depth investigation.

The EEG independent component clustering analysis generated 7 IC clusters corresponding to sources of EEG activity (Fig. S1). One of these seven clusters was located in the PCC, similarly to the fMRI results. This cluster was chosen for further analysis. A one-way ANOVA revealed a significant main effect of chanting on the power of the delta-band (1–4 Hz) [F (2,93) = 3.25, p = 0.043] (Fig. 1c). Further post hoc analysis showed significantly higher power (p = 0.011) during religious chanting (mean = 37.30; SD = 5.23) compared to non-religious chanting (mean = 33.45; SD = 7.85). The delta power of the no chanting resting state condition (mean = 35.74; SD = 4.70) did not differ from those of the two chanting conditions. The differences in power were not significant (p > 0.05) for the theta-band (4–8 Hz), alpha-band (8–12 Hz), beta-band (12–30 Hz), and gamma-band (30–45 Hz). The traditional electrode-based spectrum analysis that was computed for comparative purposes did not show any significant differences in EEG power during religious chanting, apart from a marginal trend in the vicinity of the PCC, and sporadically, in some frontal electrodes (Fig. S2).

One-way repeated measures ANOVA revealed no significant difference in mean inter-beat interval across the three conditions [F(2, 42) = 0.026; p = 0.975] (religious chanting: mean = 862.9, SD = 115.1; no chanting: mean = 862.8, SD = 108.4; non-religious chanting: mean = 860.9, SD = 111.1). ANOVA revealed significant main effects in HRV total power [TP, F(2,42) = 5.92; p = 0.005], absolute high-frequency power [HF, F(2,42) = 5.90; p = 0.006] and absolute very-low-frequency power  [VLF, F(2,42) = 4.16; p = 0.019]. Post hoc testing with Bonferroni correction revealed that compared to no chanting, religious chanting induced lower total power (p = 0.009), lower absolute high-frequency power (p = 0.014) and lower absolute very-low-frequency power (p = 0.037). Interestingly, non-religious chanting showed no significant HRV difference with the other two conditions, thereby ruling out the possibility that the EEG delta-band difference observed was due to cardiac confounds. HRV findings are summarized in Fig. 1d, and descriptive statistics are detailed in Tables 1–3. ANOVA revealed no significant difference in thoracic respiration [F(2,42) = 1.752; p = 0.186], nor in abdominal respiration [F(2,42) = 3.118; p = 0.055]. Descriptive statistics are detailed in Tables 4–5.

**Table 1:** Total Power (TP) of HRV.

Conditions	Mean (SD) of TP in ms^2^	p-value of post-hoc comparison (* denotes significant)
Religious chanting	0.0121 (0.00417)	compared to no chanting*, p = 0.009
No chanting	0.0188 (0.00920)	compared to non-religious chanting, p = 0.416
Non-religious chanting	0.0155 (0.00592)	compared to religious chanting, p = 0.164

The table shows descriptive statistics and significance values for within-group comparisons, for the total power of the heart rate variability derived from the ECG data. Repeated measures ANOVA showed a significant effect across conditions, F(2,42) = 5.92, p = 0.005.

**Table 2:** High frequency (HF) band of HRV.

Conditions	Mean (SD) of HF in ms^2^	p-value of post-hoc comparison (* denotes significant)
Religious chanting	0.00182 (0.00130)	compared to no chanting*, p = 0.014
No chanting	0.00432 (0.00421)	compared to non-religious chanting, p = 0.435
Non-religious chanting	0.00323(0.00316)	compared to religious chanting, p = 0.152

The table shows descriptive statistics and significance values for within-group comparisons, for the high frequency power of the heart rate variability derived from the ECG data. Repeated measures ANOVA showed a significant effect across conditions, F(2,42) = 5.90, p = 0.006.

**Table 3:** Very low frequency (VLF) band of HRV.

Conditions	Mean (SD) of VLF in ms^2^	p-value of post-hoc comparison (* denotes significant)
Religious chanting	0.00623 (0.00185)	compared to no chanting*, p = 0.040
No chanting	0.00823 (0.00274)	compared to non-religious chanting, p = 1
Non-religious chanting	0.00755 (0.00190)	compared to religious chanting, p = 0.085

The table shows descriptive statistics and significance values for within-group comparisons, for the very low frequency power of the heart rate variability derived from the ECG data. Repeated measures ANOVA showed a significant effect across conditions, F(2,42) = 4.156, p = 0.023.

**Table 4:** Means of Thoracic respiration.

Conditions	Mean (SD) of interval in sec
Religious chanting	3.607 (0.365)
No chanting	3.676 (0.312)
Non-religious chanting	3.572 (0.363)

The table shows descriptive statistics and repeated measures ANOVA results derived from respiratory data. No significant difference was found, F(2,42) = 1.752, p = 0.186.

**Table 5:** Mean of Abdominal respiration.

Conditions	Mean (SD) of interval in sec
Religious chanting	3.596 (0.365)
No chanting	3.714 (0.304)
Non-religious chanting	3.578 (0.355)

The table shows descriptive statistics and repeated measures ANOVA results derived from respiratory data. Results suggest a marginally significant effect across conditions, F(2,42) = 3.118, p = 0.055.

## Discussion

Combining multi-modal data, the present study illustrates neural and physiological mechanisms related to religious chanting. Religious chanting appears to increase endogenous neural oscillations in the low frequency delta-band, especially in the posterior cingulate cortex (PCC). This brain region shows the largest decrease in centrality during religious chanting in a highly-trained meditator. Such changes are not due to implicit language processes and are not attributable to differences in cardiac activity between religious and non-religious chanting. Nonetheless, religious chanting does influence cardiac activity significantly, compared to resting state.

Strong delta waves have been localized in several brain regions, including the posterior cingulate cortex, during slow-wave sleep^18^. Less strong delta oscillations are present during the awake state and have been suggested to modulate behavioral performance and memory processes^19^. Several EEG studies have found increased delta-band power due to meditation practices such as transcendental meditation^1^ and Qigong^20^. Increased delta activity in the medial prefrontal cortex was also found during Zen meditation and this may facilitate detaching from attending to one’s immediate surroundings^21^. Delta waves have also been proposed to act as inhibitory brain oscillations that prevent sources of distraction from interfering with internally focused concentration^22^.

Accumulating evidence suggest that the increased delta wave in posterior regions, especially the PCC, is related to the reduction of self-oriented thoughts^23^ and the suspension of sensory monitoring^24^. Because during Amitābha Buddha chanting individuals focus on chanting the Buddha’s name repeatedly, they experience fewer random thoughts and less mind-wandering. The increase of delta-band power in the PCC during religious chanting is a finding with important implications, especially in relation to current research efforts on meditation assisted by real-time neurofeedback^25^ and on controlling addiction craving^24^. It is worth noting that, ceteris paribus, endogenous generation of delta waves in the PCC during wakefulness would naturally lead to a de-synchronization of the PCC in relation to the rest of the brain, thereby explaining the decrease in centrality that was observed in the fMRI data. The increased delta-band power observed in the vicinity of the PCC cannot be attributed to peripheral physiological changes, as there was no difference in HRV and respiration between the religious chanting and non-religious chanting. To our knowledge, this is the first study of religious chanting or prayer that has used an active control condition (non-religious chanting), providing stricter control over confounding effects due to implicit language processing.

Religious chanting, as an active faith-based practice, overlaps with both meditation and prayer. Nevertheless, it comprises a unique, special case. Practitioners of religious chanting are encouraged to chant the name of the Buddha Amitābha as an object of meditation, while practicing the development of two equally important mental processes: samatha which corresponds to concentration and vipassana which corresponds to mindful observation^2^. During the chanting of Amitābha Buddha, the practitioner contemplates on the vow and compassion of the Buddha as a practice of vipassana. Such contemplation is meant to induce affective priming that helps the practitioner remain concentrated on the meditation object (the practice of samatha)^26,27^. By frequently practicing samatha and vipassana during religious chanting, advanced practitioners become able to combine these two aspects of the practice and reach a state called samadhi, during which both mental processes run in parallel. The present study corroborates evidence suggesting that the neurophysiological correlates of religious chanting are distinct from correlates of the extensively researched mindfulness meditation.

Mindfulness enhances alpha and theta power and in our previous EEG study on mindfulness-based stress reduction (MBSR), the alpha-band power increased while the delta-band power decreased during MBSR meditation, compared to the resting state^11^. Therefore, it appears that despite certain overlaps between mindfulness and spiritual prayer^28^, different forms of meditative practices are associated with different patterns of brain activity^29^. This implies that different religious or meditative practices may be more effective for the alleviation of specific neuropsychiatric symptoms; e.g. chanting Amitābha Buddha and relevant practices that increase delta-band power are likely to be beneficial to a wide range of patients suffering from sleep disorders. We suggest this to be the case because, apart from being the dominant frequency band during sleep^30^, delta-band power reflects the physiological tendency for sleep, by increasing due to sleep deprivation^31^ and decreasing following extended sleep^32^.

Similarly to sleep, delta-band activity has been suggested to comprise a universal response to injury or pathology^33^, due to its role for neural plasticity^30,34^ as well as for the integration of cerebral activity and homeostatic processes^33^. That is, mental states dominated by delta-band activity are considered as evolutionarily ancient states, in which compensatory and restorative mechanisms replenish biological resources in the brain and peripheral organs, resulting in beneficial effects encompassing biological and cognitive domains^33^. Variations in delta activity originate in the reticular formation^35^, which receives afferent inputs from all sensory systems and can reach the PCC through ascending, efferent connectivity, via the thalamus^36^. The loci of the PCC findings are close to the center of mass of the posterior default mode network (DMN)^37^. Although the fMRI finding is specific to the PCC, it suggests that at least part of the DMN is affected by religious chanting. Previous studies have independently shown that 53% of DMN functional connectivity variance is explained by delta-band power^38^ and that DMN activity is related to self-monitoring functions^39^. In this context, through the described mechanisms involving the suspension of modal brain activity, religious chanting appears to provide a streamlined procedure for the modulation of biological processes.

While our study on religious chanting found increased delta-band power during practice, one previous EEG study compared the Buddhist loving-kindness meditation (LKM) with Christian religious prayer and found that LKM practice was associated with increased delta, alpha and beta waves, whereas the practice of religious prayer was associated with increased alpha and gamma waves^40^. Thereby, it seems that the neurophysiological correlates of religious chanting may be different from those of meditation and religious praying. Future studies should address to what extent increased delta-band power in the PCC is common to all religious chanting (e.g. Byzantine chanting) or specific to love-kindness oriented Buddhist mantras. Moreover, it is important to investigate the extent to which specific practices (e.g. chanting, praying, meditating) can transcend cultural differences associated with specific religions.

Practitioners of Buddhist religious chanting concede that chanting Amitābha Buddha is usually accompanied by spiritual feelings of bliss and calmness, as well as visualizations of the ‘splendid Pure Land’ or ‘Land of Bliss’ referenced in the Buddhist scriptures^2^. Positive feelings and calmness nurture relaxation. This is in line with previous studies showing that prayer facilitates relaxation, which is accompanied by lower metabolism, lower breath rate and distinctive slow brain waves^41^. The emerging perspective suggests that praying may indeed counteract the physiological and psychological effects of stress and pain^42^.

In addition to the difference in brain activity, our results also showed that religious chanting can increase the stability of cardiac function, compared to the resting state. The HRV analysis is sensitive to changes in autonomic nervous system activity^43^. The reactivity of the autonomic nervous system, approximated through HRV changes, has been linked to positive and negative moods^44^. The observed decrease in HRV during religious chanting is a very plausible finding in the context of the accompanying feelings of spiritual bliss and emotional tranquility. Indeed, our previous study showed that mindfulness meditation can also induce similar cardiac effects^26^. According to the polyvagal theory, HRV indicates the modulation of cardiac activity by the autonomic nervous system and can reflect affective states, including increased stress^45^.

The different components of the HRV have been proposed to reflect modulatory effects from different sources. The VLF component and TP of the HRV were significantly lower during religious chanting, and more than half of the TP is accounted by the VLF component. The VLF component may partly reflect a fluctuation in activity of the renin-angiotensin system, which regulates the cardiovascular tone. The VLF component also reflects the peripheral chemoreceptors and the thermoregulatory mechanism^46^. The decreased VLF power during religious chanting may indicate a reduction of the cardiac defensive response and general systemic stress level. It is worth noting that the relation of HRV to cardiac vagal effects is not linear. Respiratory activity can also influence the VLF component of HRV, especially in patients with chronic heart failure^47^. However, in the present study we did not find that religious chanting had any effect on respiration.

The power of the HF component of HRV decreased during religious chanting compared to the resting state, while the HF power during non-religious chanting showed a similar trend but did not differ significantly from the HF power of the resting state. HF power is associated with respiratory sinus arrhythmia which is mainly modulated by the nucleus ambiguus activity. In fact, the HF component of the HRV reflects the magnitude of fluctuation in the modulation of cardiac activity by the parasympathetic nervous system^48^. May and colleagues have shown that a mindfulness meditation intervention can also positively modulate cardiovascular function by decreasing cardiac sympathovagal tone, ventricular workload and vascular resistance^49^.

Due to the monotonous repetition of the brief chant and the ability of experienced meditators to effectively enter the meditational mental state at will, the associated brain activation is assumed to be relatively homogeneous across a meditative session, especially when lasting only a few minutes. Therefore, the EEG and fMRI versions of the paradigm can be considered equivalent, despite minor differences in the length of experimental trials between the fMRI conditions and between the two neuroimaging modalities. A possible limitation of the present study is that the fMRI and EEG data were acquired from different subjects, with considerable differences in religious chanting experience. For future research, we hypothesize that replicating the present study with a sample comprised of Buddhist monastics would yield similar results with stronger effect sizes, whereas replicating the present study with a sample comprised of non-believers would result in no significant difference between chanting conditions.

The lack of cognitive and affective ratings, with regards to either the effect of each experimental condition or the overall psychometric evaluation of each participant, is another potential shortcoming of the present study. Future studies should include the acquisition of such data, with regards to which we hypothesize that the effects reported herein would correlate positively with positive affect and negatively with the level of self-referential cognition. Further, we recommend that future studies acquire EEG data in an electromagnetically shielded EEG cabin, with a high sampling rate (e.g. 5000 Hz), to enable analysis of high gamma band activity as well.

In conclusion, compared to non-religious chanting, the PCC decreases in centrality due to a regional increase in endogenous generation of delta oscillations. These functional effects are not due to peripheral cardiac or respiratory activity, nor due to implicit language processing, and are associated to feelings of transcendental bliss and decreased self-oriented cognition. Compared to the resting state, religious chanting increases the stability of cardiac activity, reflecting enhanced stability in the regulation of cardiovascular tone and the parasympathetic modulation of cardiac function. Such physiological changes illustrate the mechanisms through which relaxing meditative practices exert positive stress-reducing effects. The neurophysiological correlates of religious chanting are somewhat different from those of mindfulness meditation and those of other types of religious prayer and future research should address the replicability and specificity of the neurophysiological effects of different religious and meditative practices, as well as their differential suitability as psychological interventions. Research in this field is still in a nascent stage and the tentative interpretations offered here can serve to provide several hypotheses for future research.

## Materials and Methods

### Participants

Twenty-two participants with at least one year of meditative experience in religiously chanting Amitābha Buddha, for at least 15 minutes per day, participated in the EEG/ECG study. The age range was 40 to 52 years old (mean = 46.5; SD = 2.6; 12 females). One participant’s ECG data were usable but his EEG data were dropped due to operational error. One additional 43-year old female participant, who was a highly trained meditator by virtue of being a Bhikṣuṇī Buddhist monastic, participated in an fMRI case study measurement using the same paradigm. All participants were free from any neurological, psychiatric or other mental disorder. Ethical approval was granted by the Human Research Ethics Committee of the University of Hong Kong, and this research was performed in accordance with relevant guidelines/regulations. All participants signed an informed consent form prior to participation in the experiment.

### Procedure

The experimental paradigm consisted of two parts. The first part featured a perceptual task that was published elsewhere^26^, and only the second part is described herein. All participants underwent three conditions in random order. The experimental condition consisted of mentally chanting Amitābha Buddha (religious chanting condition); that is participants chanted internally, silently and without any movement of the lips. It is worth noting that this type of internal, silent chanting is also a relatively common Buddhist practice, particularly when meditative moments are sought while being in public places (e.g. while waiting at an airport or train station). Resting state without chanting was used as a control condition (no chanting condition), similarly to a previous study^13^. During resting state, subjects maintained their eyes closed, similarly as during chanting. Additionally, to control for the potential effect of implicit language processes, a second control condition was employed, featuring mental chanting of the Santa Claus (non-religious chanting condition). Both chants comprised of four Chinese characters (阿彌陀佛 and 聖誕老人) and were comparable in terms of linguistic complexity. Approximate phonological representations of the two chants were [æ mʃ tæ fæ], corresponding to “Amitābha (Buddha)” and [ɪɛn dæn laʊ rɛn] corresponding to “Santa Claus old man”. Both figures referred to in the chants are generally considered as supernatural and carry solely positive connotations, even for atheists, in popular culture, thereby controlling the objective affective content across the two conditions. EEG data analysis featured standard preprocessing, independent component analysis, and independent component clustering. FMRI data were used for eigenvector centrality mapping^50^. ECG and respiratory data were subjected to heart rate variability analysis.

### EEG data

EEG data were acquired during all conditions in a quiet EEG laboratory room, in the presence of one researcher, using a 128-channel EGI^TM^ system (Electrical Geodesics, Inc. USA). The impedances of all electrodes remained below 30 KΩ, in accordance with the requirements of the EGI system. The sampling rate of the EEG data was 1000 Hz. Each EEG condition, including the resting-state lasted 10 minutes. EEG data were processed and analyzed using the EEGLAB toolbox^51^ (https://sccn.ucsd.edu/eeglab/) and Matlab 11.0 (MathWorks Inc. USA). In the preprocessing stage, the EEG data were resampled to 250 Hz and band-pass filtered by a finite impulse response filter between 0.1–100 Hz. To reduce artifacts caused by alternating current, data were additionally notch filtered by a short nonlinear infinite impulse response filter with a band-stop range between 47–53 Hz. Following visual inspection, segments of low data quality due to artifacts were deleted, while bad channels were reconstructed using spherical interpolation of the signals. Independent Component Analysis (ICA) was used to remove components due to eye movement, blinking, and other artifacts. Data were reconstructed from the retained components.

Using EEGLAB, the similarity of independent components (ICs) was estimated and similar components were grouped together into functionally equivalent clusters. Comparisons between conditions were based on major clusters rather than single channels. First, for each subject, ICA was performed on the data from each condition to generate ICs. Then, the dipole locations of each IC were generated using the DIPFIT2 (EEGLAB plug-in) function. IC clusters were generated by *k*-means clustering based on similar dipole location (weight: 2/3) and power spectrum (weight: 1/3). The weight distribution was chosen based on the assumption that functionally similar components should have similar spatial location and power activity. The outlier threshold was set at 2.5 standard deviations (SD). The clustering procedure was repeated more than 10 times, each time with a different *k* parameter setting. Six to twenty clusters were generated and the *k* parameter setting that generated the most distinctive clusters was identified, ensuring that the clusters were consistently generated under different *k* parameter settings. This procedure finally resulted in seven IC clusters. Spectrum analysis and a one-way ANOVA were performed on all major frequency bands, using the ICs from a specific cluster of interest (as well as using the traditional electrode channel approach, for comparative purposes).

### MRI data

To identify ICs that are most influential, the same paradigm was used to acquire fMRI data from an additional participant with long-term experience of religious chanting by virtue of being an experienced Buddhist monastic. Scanning was performed with a 3.0 T Philips MRI scanner. First, a T1-weighted scanning sequence was acquired, featuring the following parameters: FoV = 256 × 150 × 240 mm, acquisition matrix = 256 × 256, TR = 15 ms, TE = 3.26 ms, flip angle = 25°, slice thickness = 1.5 mm, number of slices = 100, voxel resolution (x,y,z,) = 0.94 × 1 × 1.5 mm. FMRI images were obtained with gradient echo-planar imaging (EPI), with an 8-channel SENSE head coil, featuring FoV = 230 × 140 × 230 mm, acquisition matrix = 64 × 64, TR = 2000 ms, TE = 30 ms, flip angle = 90°, number of slices = 32, slice thickness = 3 mm, and slice gap = 1.5 mm. In total, 688 dynamic volumes were acquired, comprising the 3 conditions: religious chanting (243 dynamic volumes; duration 8.1 minutes), non-religious chanting (243 dynamic volumes; duration of 8.1 minutes) and resting state (202 dynamic volumes; duration 6.7 minutes). The resting-state acquisition was shorter due to scanning time constraints and because the resting-state condition was only meant to be used as a neutral baseline, to establish the directionality of any potentially ambiguous findings.

FMRI data were processed using the Leipzig Image Processing and Statistical Inference Algorithms^52^ (LIPSIA; version 2.2.7 – released in May 2011). Pre-processing consisted of slice time correction, movement correction, spatial smoothing with FWHM = 6 mm, affine non-linear normalization to MNI space, and regression of covariates of no interest (global mean and movement parameters). Eigenvector centrality mapping (ECM) was used to investigate whole-brain functional connectomics. ECM is a graph theory method, which can identify the most influential nodes of a network^50^. The long and continuous conditions of the paradigm were particularly suited for ECM, which was applied similarly to a previous study on the neural correlates of emotion elicited through continuous musical stimulation^53^. One ECM map was computed for each of the two chanting conditions (i.e. religious chanting and non-religious chanting) and the two conditions were compared via a 1^st^-level contrast. The resulting ECM difference map was incrementally thresholded until only the region with the greatest difference in ECM between the two conditions remained (|z| > 0.85). The explorative results of the fMRI analysis were used to confirm the validity of the Independent Component Clustering results, as well as to guide the selection of a specific IC cluster for more detailed investigation.

### ECG and respiratory data

Physiological data of cardiac and respiratory activity were collected using the ADInstrument’s PowerLab system (www.adinstruments.com/products/powerlab). Breath intervals, representing thoracic and abdominal respiratory activity, were computed for each participant and each condition, using Matlab. Raw ECG data were cleaned via a Butterworth band pass filter and the inter-beat-interval (IBI) was extracted following the replacement of outliers (3 standard deviation away from the mean) via spline interpolation. Using the HRVAS toolbox (https://sourceforge.net/projects/hrvas), the IBI data were detrended and the time/frequency domain features of the HRV were computed. Frequency ranges for the VLF, LF, and HF bands were set to 0–0.04 Hz, 0.04–0.15 Hz, and 0.15-0.4 Hz respectively. The power of these frequency bands was estimated using the Lomb-Scargle periodogram. Derived HRV metrics were subjected to statistical testing using SPSS 24.0. One-way repeated measures ANOVA and post hoc tests (where applicable) were used to assess differences between conditions. The alpha level of significance was set at 0.05.

## Supplementary information

Supplementary file

## Data Availability

The datasets generated during and/or analysed during the current study are available from the corresponding author on reasonable request.
