# Supplementary material for: The neurophysiological correlates of religious chanting
Source: Sci Rep. 2019 Mar 12;9:4262. doi: 10.1038/s41598-019-40200-w (PMC6414545; doi:10.1038/s41598-019-40200-w)
Supplement: Supplementary file 1 — Supplementary file [file 41598_2019_40200_MOESM1_ESM.docx]

**The neurophysiological correlates of religious chanting**

**Junling Gao^1^, Hang Kin Leung^1^,** **Bonnie Wai Yan Wu^1^, Stavros Skouras^2^, Hin Hung Sik^1*^**

^1^Buddhism and Science Research Lab, Centre of Buddhist Studies, The University of Hong Kong, Pokfulam, Hong Kong

^2^Department of Biological and Medical Psychology, Faculty of Psychology, University of Bergen, Bergen, Norway

*** Correspondence:**

Hin Hung Sik

hinhung@hku.hk

**Keywords: religious chanting, EEG, neurophysiology, eigenvector centrality, source localization, independent component clustering, HRV.**

**Supplementary material**


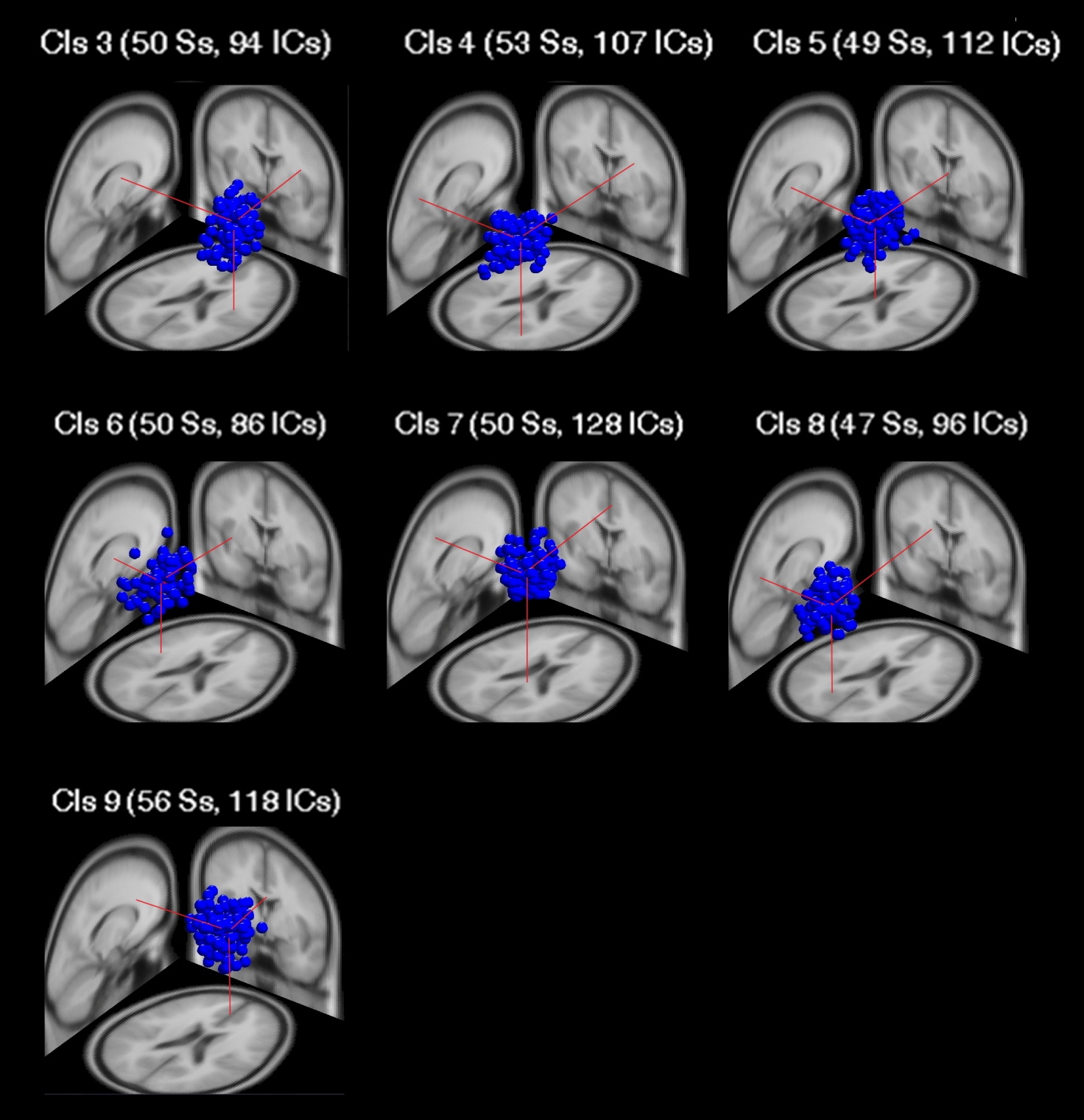


**Figure S1. Results of cluster analysis.**

The figure shows the seven clusters of independent components that were identified across participants using EEGLab.


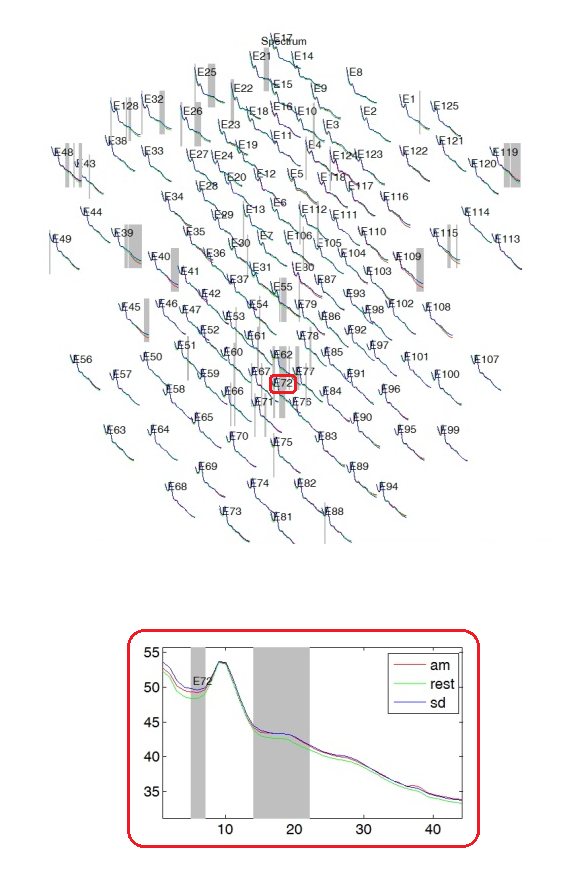


**Figure S2. The traditional EEG electrode-based approach.**

The upper figure shows the overall channel-based spectrum analysis (upper figure). The lower figure shows the spectrum analysis on channel E72. Shadows indicate significant differences at the covered spectrum among the three conditions (ANOVA).
